# The Role of Left Hemispheric Structures for Emotional Processing as a Monitor of Bodily Reaction and Felt Chill – a Case-Control Functional Imaging Study

**DOI:** 10.3389/fnhum.2016.00670

**Published:** 2017-01-06

**Authors:** Viktoria Grunkina, Katharina Holtz, Kai Klepzig, Jörg Neubert, Ulrike Horn, Martin Domin, Alfons O. Hamm, Martin Lotze

**Affiliations:** ^1^Functional Imaging Unit, Center of Diagnostic Radiology and Neuroradiology, University of GreifswaldGreifswald, Germany; ^2^Department of Psychology, University of GreifswaldGreifswald, Germany

**Keywords:** insula stroke, chill response, skin conductance response, emotional processing, music

## Abstract

**Background:** The particular function of the left anterior human insula on emotional arousal has been illustrated with several case studies. Only after left hemispheric insula lesions, patients lose their pleasure in habits such as listening to joyful music. In functional magnetic resonance imaging studies (fMRI) activation in the left anterior insula has been associated with both processing of emotional valence and arousal. Tight interactions with different areas of the prefrontal cortex are involved in bodily response monitoring and cognitive appraisal of a given stimulus. Therefore, a large left hemispheric lesion including the left insula should impair the bodily response of chill experience (objective chill response) but leave the cognitive aspects of chill processing (subjective chill response) unaffected.

**Methods:** We investigated a patient (MC) with a complete left hemispheric media cerebral artery stroke, testing fMRI representation of pleasant (music) and unpleasant (harsh sounds) chill response.

**Results:** Although chill response to both pleasant and unpleasant rated sounds was confirmed verbally at passages also rated as chilling by healthy participants, skin conductance response was almost absent in MC. For a healthy control (HC) objective and subjective chill response was positively associated. Bilateral prefrontal fMRI-response to chill stimuli was sustained in MC whereas insula activation restricted to the right hemisphere. Diffusion imaging together with lesion maps revealed that left lateral tracts were completely damaged but medial prefrontal structures were intact.

**Conclusion:** With this case study we demonstrate how bodily response and cognitive appraisal are differentially participating in the internal monitor of chill response.

## Introduction

Chill can be experienced in various ways (shivers down the spine, piloerection, or lump in the throat) and is often accompanied by measurable changes in various physiological response systems (Panksepp, 1995). Increased sympathetically mediated sweat gland activity can be observed in most chill reactions, thus phasic changes in skin conductance is used as a reliable indicator of the physiological arousal typically associated with a chill response (Craig, 2005; Guhn et al., 2007; Grewe et al., 2009; Salimpoor et al., 2009).

Chill experiences often emerge in response to pleasant music (e.g., McCrae, 2007) but can also be observed in response to dissonant auditory stimuli (Dellacherie et al., 2011). Although the chill response is often used to investigate emotional response to music (Nusbaum and Silvia, 2011), only few studies have investigated neural networks that are involved in regulating the chill response evoked by music. Using positron emission tomography, Blood and Zatorre (2001) demonstrated that listening to individually selected chill provoking music leads to increased activation of the PFC and the AIC. Interestingly, the AIC has a profound role in monitoring bodily response and is therefore important for interoception (Critchley et al., 2004; Wiens, 2005; Singer et al., 2009). Furthermore, it has structural interactions to other emotional response processing areas (Dupont et al., 2003). Accordingly, increased activation of the AIC has been reported during anticipation of aversive and painful stimuli (Friebel et al., 2011; Holtz et al., 2012). In contrast, the PIC seems to be involved in rather perceptual discriminative tasks of the aversive stimulus itself, like processing of intensity and lateralization of pain (Brooks et al., 2002; Craig, 2009). A linkage between emotional processing and autonomic response is conducted via the insula as demonstrated with associations between insula activation and autonomic regulation parameters in a meta-analysis (Beissner et al., 2013). Overall, large meta-analyses on different imaging studies have demonstrated predominantly four subregions in the insula: cognitive, social-emotional, sensory, and sensorimotor (Kurth et al., 2010). Studies on patients with unilateral lesions of the insula have demonstrated that especially left hemispheric damage results in deficits in the emotion of disgust (Calder et al., 2000; Straube et al., 2010), decrease of the motivational dimension for stimuli assessed to be highly rewarding prior to the lesion (Naqvi et al., 2007), but also hypersensitivity to pain (Starr et al., 2009).

The PFC has been reported to be associated with emotional control (Eippert et al., 2007), executive function and contributes together with the parietal lobe to working memory processes (Cohen et al., 1997; Owen, 1997; Rottschy et al., 2012). Within the PFC Brodmann Areas 46 and 47 (BA 46/47) are known to process emotional valences for stimuli of the visual (Lotze et al., 2006) or the auditory domain (Wildgruber et al., 2005). Besides the highly lateralized role for semantic and phonological processing role (Poldrack et al., 1999) or the processing of prosody (Wildgruber et al., 2005), the lateral PFC regions labeled as BA 44/45 also have multimodal connections especially from auditory association cortices (Petrides and Pandya, 2002). The more dorsal regions of the PFC (BA 8/9; posterior medial frontal cortex) are associated with working memory core processes (Rottschy et al., 2012), involved for instance in the cognitive component of aesthetical evaluation of stimuli (Cela-Conde et al., 2004). All prefrontal structures therefore may serve for differential emotional-cognitive evaluation of musical stimuli involved in a chill response.

Both areas, the PFC and the AIC, are anatomically intensely interconnected (Dupont et al., 2003). The versatile projections and functions of both regions indicate a complex interplay of cognitive and emotional processes.

We here investigated the response to auditory chill inducing stimuli in a patient with left insula lesion who verbally communicated an animating effect of musical chill. The patient experienced a complete medial cerebral artery infarct 7 years before investigation (chronic stable state) damaging left sided insula together with the ventrolateral PFC, the anterior temporal lobe and the left thalamus. We investigated both the SCR and the subjective chill experience during listening to high chilling auditory stimuli while performing fMRI and compared her data with data of an age, gender and intellectual abilities precisely matched HC. We here used pleasant and unpleasant chill stimuli in order to enable a differentiation of behavior, psychophysiology or functional representation. fMRI was used to investigate possible chill associated functional representation. High resolution structural T1, T2-weighted and diffusion weighted imaging served as the demonstration of the lesion of gray and white matter structure. According to previous reports on AIC and PFC function we expected a mismatch between cognitive evaluation of emotional chill and bodily response, which should be referred to by structural lesion and functional mapping of emotional response representation.

## Method

### Participants

**FIGURE 1:**
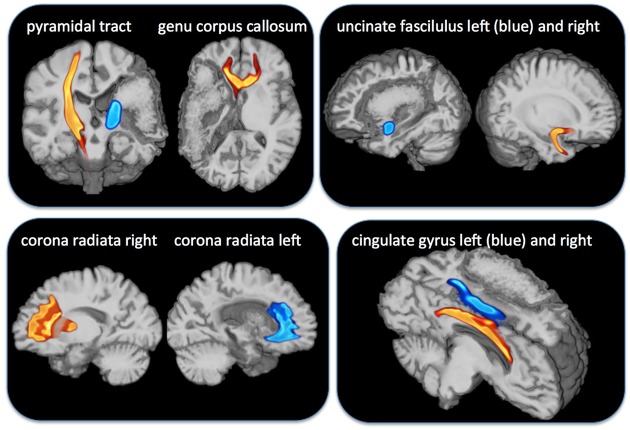
**Overlay of diffusion weighted imaging for tracts indicated above and the lesion map of the patient MC with a complete damage of the medial cerebral artery area.** The pyramidal tract (**Top**, outer left) and the genu of corpus callosum (**Top**, inner left) were completely damaged on the affected left side. The uncinate tract (**Top** right) was also completely damaged on the affected side but the corona radiate (**Bottom** left) and the cingulate gyrus (**Bottom** right) were left intact.

We investigated a 47-year-old, left-handed participant (MC) with an extended stroke of the left middle cerebral artery. The stroke occurred 7 years before fMRI-investigation and the lesion has a volume of 260 cm^3^. White matter tracts such as the pyramidal tract and the uncinate fasciculus were no more verifiable using diffusion weighted tractography (DTI, see **Figure 1**). She suffered from Broca aphasia, neglect on the right hemifield and a hemiplegia including an upper limp plegia and lower limb paresis.

We had the opportunity to compare MC with a healthy woman (HC), who had a very similar demographic, social, and intellectual background. Being born in the same year and working in the same profession with a similar level of career as MC, when she experienced the stroke, it makes her the perfect comparator.

### Materials, Design, and Procedure (for Details Please Refer to Supplementary Information)

To evoke pleasant and unpleasant chill reactions we presented musical stimuli, which showed a high ability to induce chills (Guhn et al., 2007) and recordings of harsh sounds (prepared for this study). In accordance with Cox (2008) harsh sounds were created by scraping objects and materials (duration 7.5–9.5 s; digitally embedded into classical music pieces; see Supplementary Information).

MC and HC were instructed to listen carefully to the music and sounds and to report intensity, onset, and duration of each chill experience by pressing a foam covered handle device with their right hand. Then they were placed in the scanner and equipped with headphones. The whole experiment lasted about 30 min. Whereas SCR is the optimal psychophysiological parameter for measuring bodily response to chills, heart rate does not reliably change during chill response (Guhn et al., 2007). Therefore, only the sympathetic aspect of autonomous regulation seems to be associated with bodily response to chill.

### MRI Measurement

Images were collected with a 3 T Magnetom Verio (Siemens, Erlangen, Germany) using a 12-channel head coil. Echo-planar imaging (864 volumes with 34 slices, temporal resolution 2 s), phase and magnitude images to calculate a fieldmap aiming at correcting geometric distortions, T1-weighted high resolution anatomical imaging and diffusion weighted imaging were performed over the whole head volume.

### Imaging Processing and Statistical Analyses

Data of the handle device and skin conductance were processed with Brain Vision Analyzer 2.0 (Brain Products, Gilching, Germany). Skin conductance data were down-sampled to 10 Hz and differentiated into tonic and phasic activity using Ledalab toolkit (Benedek and Kaernbach, 2010). Analysis of SCR was focused on chill passages, which were defined as 10 s windows starting either with the onset of the harsh sounds (unpleasant chill hotspot) or with the beginning of specific passages in the music stimuli (pleasant chill hotspot). SCR was then scored as the average phasic activity (Windel et al., 2015) in a 9 s window starting 1 s after the onset of the chill passage.

### MRI Data

Lesion volumes were calculated on the basis of the T1-weighted image. Diffusion weighted data were processed utilizing DTIFIT, BEDPOSTX and PROBTRACKX of the FSL software package. Seed masks for tractography were created from the activation maxima of the fMRI statistical maps within the PFC using a spherical kernel with a diameter of 15 mm to ensure the inclusion of white matter. We also applied additional seed masks for the evaluation of the arcuate fasciculus, cingulate gyrus, the posterior limb of the internal capsule, the genu of the Corpus Callosum and the Corona radiata (Mori et al., 2005).

FMRI data were analyzed using the SPM8 software (Wellcome Department of Cognitive Neurosciences, London). Spatial pre-processing included realignment to the first scan, unwarping, coregistration to the T1 anatomical volume images. T1 images were spatially normalized using the New Segment function of SPM 8 and DARTEL (Ashburner, 2007) and functional were normalized to this template into the MNI-space. Normalized functional images were smoothed (6 mm). fMRI data were analyzed using a general linear model (GLM) with chill condition versus baseline condition (bird song). At the first level of analysis, a fixed-effect analysis was performed to obtain fMRI-activation maps for each subject due to pleasant and unpleasant stimuli. Onsets were chosen individually for MC and HC based on the device pressure and events were modeled 10 s around this time point. We used a statistical threshold of *p* < 0.05 corrected for multiple comparisons in the whole brain volume (FWE).

For HC we further conducted two psychophysiological interaction (PPI) analyses with SPM8 to investigate, which voxels in the brain increase their interaction with the left and the right AIC during a chill event. Therefore the convoluted time courses (task × seed) were analyzed in a GLM while the task time course and the seed time course itself were included as regressors.

## Results

Most of the applied harsh sounds resulted in chill reports from both participants. Whereas HC reported chills in response to music (chill intensity: 0.12 ± 0.06) and harsh sounds (0.11 ± 0.09) with similar intensities, MC reported higher intensities for chills in response to harsh sounds (0.16 ± 0.08) than for chills in response to music (0.09 ± 0.07).

**FIGURE 2:**
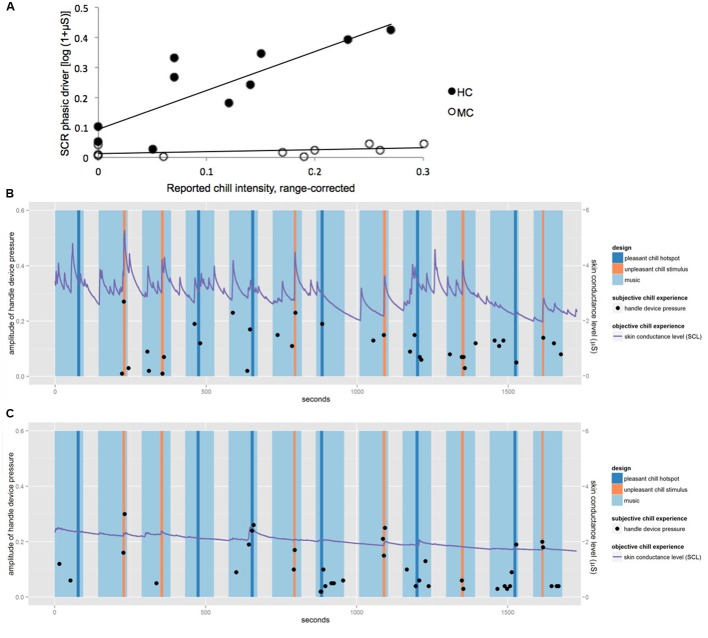
**(A)** Correlation between reported chill intensity and SCR for the 10 chill passages for HC and MC. The linear association is represented by lines, positive correlation for HC (*r* = 0.86, *p* < 0.01) and no relevant association for MC (*r* = 0.53, n.s.). **(B)** Skin conductance level (SCL) and chill reports for HC. **(C)** SCL and chill reports for MC.

**Figure 2A** shows the correlation between SCR and the reported chill intensities for the 10 chill passages for MC and HC. For HC the reported chill intensity was positively correlated with SCR (*r* = 0.86, *p* < 0.01), whereas for MC no relevant association was observed (*r* = 0.53, n.s.). **Figures 2B,C** show skin conductance level (SCL) and chill reports over the course of the experiment for HC and MC. Though both participants reported chills at a similar range of intensity throughout the entire experiment only HC showed associated autonomic responses regularly. MC showed an attenuated activation of the sympathetic nervous system as expressed in SCR compared to HC.

**FIGURE 3:**
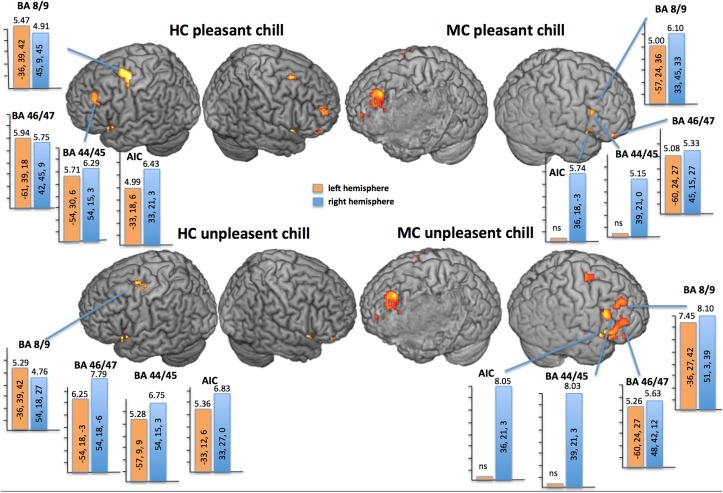
**fMRI-effects of the contrast chill events versus non-chilling birdsong (*p* < 0.05; FWE-corrected for the whole brain) of each participant overlaid on the individual segmented brain.** Bars show the highest *t*-value within ROIs (AIC; BA 44/45; BA 46/47 and BA 8/9) for each condition and participant [HC and the MCA left lesion patient (MC)]. **Top**: fMRI-response to pleasant chill (music); **Bottom**: fMRI-response to unpleasant chill (scratches). MC showed no relevant left hemispheric fMRI response in MCA for AIC and BA 44/45 but right hemispheric increase. HC showed balanced fMRI-activation within ROIs between hemispheres.

Unpleasant and pleasant chill stimuli evoked fMRI-activations against baseline in different areas of the PFC and temporal cortices, as well as the S2, the AIC, the A1, the thalamus, and bilateral amygdala in both participants. More specifically for the PFC and the AIC we found quite balanced activation for both hemispheres for HC but for MC lateral PFC areas such as AIC and BA 44/45 showed only relevant activation in the right intact hemisphere (**Figure 3**). Remarkably, not only the pleasant chills were associated with bilateral ventral striate activation in HC (left: *t* = 4.71; *p*_FWE_ = 0.008; right: *t* = 5.72; *p*_FWE_ = 0.001) but also the unpleasant chills showed a right hemispheric ventral striate fMRI-activation (*t* = 5.30; *p*_FWE_ = 0.001). For MC the pleasant chills were not associated with relevant ventral striate activation, but unpleasant chills showed bilateral ventral striate activation (left: *t* = 3.89; *p*_FWE_ = 0.014; right: *t* = 3.66; *p*_FWE_ = 0.03).

When investigating the structural interaction between the lateral PFC-fMRI-activation sites and other areas we found no relevant interaction of the damaged left hemisphere in the patient. In the HC the lateralization index (LI) of the fractional anisotropy values (FA) of the dlPFC seed was on average (individual seed for pleasant and unpleasant chill) on -0.07 and for the vlPFC -0.03. In contrast, LIFA of the patient was strongly lateralized to the right hemisphere (dlPFC: 0.38; vlPFC: 0.64).

A PPI (**Table 1**) performed for the HC for both pleasant and unpleasant chills demonstrated that the right AIC was interconnected with the bilateral Heschl gyrus but not to prefrontal ROIs (BA 44–47; BA 8/9). In contrast, the left AIC was not only interacting with the bilateral Heschl gyrus but also with the bilateral prefrontal ROIs (right BA 44/45, left BA 46, right BA 47, and right BA 9).

**Table 1:** Psychophysiological interaction performed for the HC for pleasant and unpleasant chills.

Interconnection of		Left hemisphere			Right hemisphere		
		*t*	*p*_FWE_	Coordinates (*x*, *y*, *z*)	*t*	*p*_FWE_	Coordinates (*x*, *y*, *z*)
AIC right	Heschl gyrus	3.68	0.007	-36, -33, 12	3.82	0.004	42, -27, 9
	BA 44–47		n.s.			n.s.	
	BA 8/9		n.s.			n.s.	
AIC left	Heschl gyrus	3.22	0.03	-36, -33, 12	4.37	0.001	42, -27, 9
	BA 44/45		n.s.		3.99	0.008	8, 24, 9
	BA 46	4.13	0.001	-45, 30, 21		n.s.	
	BA 47		n.s.		3.81	0.02	51, 21, 0
	BA 9		n.s.		4.35	0.004	48, 9, 36

## Discussion

We examined the perception of pleasant and unpleasant chills in a patient with an MCA stroke in the left hemisphere including the left insula and ventral parts of the PFC. In comparison to a healthy age, gender and profession matched control (HC) we found a comparable subjective chill appraisement but a selective decrease in objective chill parameters (SCR) for both unpleasant and pleasant chill stimuli for the patient. Correspondingly, SCR and assessment scores were positively associated in HC but not in MC. Diffusion imaging discovered selective destruction of more lateral structures such as the uncinate fasciculus whereas medial structure such as the radiate corona and the cingulate were undestroyed. Psychophysiological differences between participants went along with changes in functional representation during listening to stimuli. In MC AIC and BA 44/45 were only relevantly activated in the right hemisphere, but more anterior representation in BA 8/9 and 46/47 was balanced between hemispheres. We interpreted the findings that intact right hemispheric areas for auditory recognition and intact working memory allowed for an adequate evaluation of the stimuli presented but damaged left hemispheric AIC diminished bodily response.

We used musical stimuli, which fulfill chill criteria in most healthy participants (Grewe et al., 2005; Guhn et al., 2007). We furthermore created a set of stimuli with chills inducing aversive events using scratching sounds, which have previously been described to evoke avoidance reaction in humans (Halpern et al., 1986). These sounds have already been rated as more reliably evoking chills than musical sounds (Grewe et al., 2011).

Whereas the onsets and duration of aversive chills were constant between participants, those of pleasant chills differed between individuals, which makes an individualized fMRI-design necessary. In HC objective and subjective chill parameters were highly positively associated but dissociated in MCA.

Our stimuli not only reliably evoked SCR-changes in HC, they also activated brain areas that have reported in previous functional imaging studies on chill response. Interestingly, in HC not only pleasant chills were associated with bilateral ventral striate response as reported previously (Blood et al., 1999) but also unpleasant chills. The ventral striatum has been associated not only to rewarding emotions but has also been linked to the anticipation of aversive events in a tight interaction with the orbitofrontal cortex (Bolstad et al., 2013). This ventral striate response to unpleasant stimuli could also be observed in our MCA-patient, supporting the view that the harsh sound were processed as unpleasant stimuli in both participants.

The case study at hand highlights the important role of the left AIC for chill experience. With respect to musical stimuli the anterior insula has been especially active during the processing of unexpected chords (Koelsch et al., 2005). Overall, the anterior insula is directly associated with multimodal emotional stimuli processing (Craig, 2011).

A loss of feeling for music after left AIC lesion has already been described (Griffiths et al., 2004) as well as loss of craving for smoking (Naqvi et al., 2007). However, the question arises whether left AIC is specific for the loss of bodily response to chills as measured here by monitoring changes in skin conductance. Very roughly, changes in skin conductance are indirectly associated with emotional processing and the AIC is one of the key areas involved. Both SCR and AIC activation magnitude are modulated by attention and anticipation (Bonson et al., 2002; Straube and Miltner, 2011). Overall, the left AIC is highly important in orchestrating emotional expression as indexed by increase in autonomic arousal. However, with respect to the lateralization of insula representation for autonomic response, a recent ALE meta-analysis on fMRI-studies has weakened the hypothesis of a left/right dichotomy in the insula (Craig, 2005) for the specialization in autonomic regulation (Beissner et al., 2013).

We would predict that a loss of bodily response to chilling stimuli is restricted to lesions of the left AIC and will not be present for patients with right AIC lesions, which has to be proven in a study testing groups of right and left anterior insula damaged patients with our paradigm.

We showed that MC activated the right AIC and the right BA 44/45, whereas BA 46/47 and BA 8/9 were bilaterally involved. The loss of autonomic changes during perceived chill experiences in this patient suggests that embodiment of this emotional experiences to chills is processed in a network including left AIC and BA 44/45. Lesions within this network seem to reduce the craving response in anticipation of a drug (Naqvi et al., 2007) or the emotional embodiment in response to music (Griffiths et al., 2004).

Nevertheless, the appropriate verbal report of a chill experience in MC is noteworthy. The intact regions are therefore critical for an appraisal of the stimulus regardless of the physiological sensation. It might be that intact prefrontal areas allowed for an evaluation of the music but diminished left hemispheric insula function and destroyed connections with lateral prefrontal areas abolished the association between bodily response and chill experience. Especially in BA 46/47, evaluation of emotional valence has been repetitively reported both for visual and acoustic stimuli (e.g., Wildgruber et al., 2005; Lotze et al., 2006). Furthermore, the intact dorsal regions BA 8/9 are associated with working memory processes and involved in the aesthetical evaluation of stimuli (Cela-Conde et al., 2004), which probably contribute to the reported chill impression in MC. Whether MC experienced other physiological responses independent from the SCR, which could have been driven an interoceptive process and resulting chill experience cannot be elucidated in detail.

It has to be mentioned that the AIC is only one major part of a network monitoring bodily responses and interoception (e.g., Critchley et al., 2004). Connectivity studies described a salience network (e.g., Menon and Uddin, 2010) with AIC as a hub whereas lateral prefrontal and lateral parietal areas are tightly interconnected in an executive network.

Therefore, lateralized chill response might well be processed in interactions between the AIC and the PFC, but not in single areas. This is underlined by severe structural damage present in our patient for lateral fiber tracts connecting emotional processing and prefrontal cognitive appraisal of stimuli. Our functional interaction analysis (PPI) underlined the network difference between left and right hemispheric interactions of the AIC: in HC only left AIC interacts with the PFC whereas the right insula was not interacting with PFC in our paradigm.

### Limitations

This case-control study is only a first step for investigating chill processing for positive and negative emotional assessed auditory material. The patient investigated here showed a complete left side MCA damage including various other areas besides the left AIC. Lesion mapping in a number of patients is necessary in order to specify the role of the left AIC using the same chill parameters and investigating fMRI-response in other areas of the prefrontal lobe. This investigation should again integrate diffusion weighted imaging to define structural intactness of interaction between areas. Up to now the conclusions of this study are very limited and do not allow for specifically pointing to the role of the left AIC for the processing of chill.

## Conclusion

Whereas left hemispheric lesion after MCA stroke abolished physiological expression (indexed by SCR and SCL-changes) or embodiment of the chill responses, the evaluation of the chill experience was left unchanged. We hereby highlighted the role of left hemispheric limbic areas for emotional response expression and monitoring. Nonetheless, prefrontal regions probably generated a chill experience that could be reported using an analog rating-device in the described patient. Further lesion studies will help to investigate the specific role of each of these areas.

## Ethics Statement

This study was carried out in accordance with the recommendations of the Ethics Committee of the German Society of Psychology. All subjects gave written informed consent in accordance with the Declaration of Helsinki. The protocol was approved by the Ethics Committee of the German Society of Psychology (DGPS; ML_012015_rev).

## Author Contributions

VG helped with measurement, data evaluation and writing of the manuscript. KH worked on study design and writing of the manuscript. KK helped with measurement, data evaluation and writing of the manuscript. JN helped with measurement, data evaluation and writing of the manuscript. UH helped with data evaluation and writing of the manuscript. MD helped with study design, data evaluation and writing of the manuscript. AH worked on the study design and writing of the manuscript. ML worked on the study design and writing of the manuscript.

## Conflict of Interest Statement

The authors declare that the research was conducted in the absence of any commercial or financial relationships that could be construed as a potential conflict of interest.

## Abbreviations

A1: primary auditory cortex
AIC: anterior insula cortex
BA 44/45: Broca’s area
BA: Brodmann’s area
fMRI: functional magnetic resonance imaging
FWE: family wise error
HC: healthy control
MC: patient with complete medial cerebral artery infarction left
MCA: medial cerebral artery
PFC: prefrontal cortex
PIC: posterior part of the insula cortex
S2: secondary somatosensory cortex
SCR: skin conductance response
