# Supplementary material for: The Role of Left Hemispheric Structures for Emotional Processing as a Monitor of Bodily Reaction and Felt Chill – a Case-Control Functional Imaging Study
Source: Front Hum Neurosci. 2017 Jan 6;10:670. doi: 10.3389/fnhum.2016.00670 (PMC5216041; doi:10.3389/fnhum.2016.00670)
Supplement: Supplementary file 1 [file Data_Sheet_1.DOCX]

**Supplemental Methods**

***Participants***

MC showed a complete left MCA infarct including the lateral left frontal lobe, the complete left insula, all parts of the left basal ganglia, the thalamus, parts of the temporal lobe, the hippocampus and the amygdala. The stroke occurred 7 years before fMRI-investigation and the lesion has a volume of 260cm^3^. A healthy age matched women (HC), who had a very similar demographic, social and intellectual background served as a control subject.

***Data measurement***

*Sound Stimuli*

Stimulus length was adjusted to 95 seconds. Together with 7 seconds of silence, which followed subsequently, the total duration of each experimental trial was 102 seconds. As baseline we selected 35 seconds of bird song followed by 7 seconds of silence resulting in a total length of 42 seconds.

In order to increase the susceptibility to chill reactions (Ely, 1975; Reuter and Oehler, 2011) the general descriptions of the sounds were presented visually (composer, harsh sound material and bird song baseline) projected on a tilted mirror mounted on the head coil and visible throughout the entire stimulus presentation.

*Pleasant chill evoking music pieces and chill passages according to Guhn et al. (2007)*

| Music piece | Chill passage (pleasant chill hotspot) |
| --- | --- |
| Tomaso Albinoni “Adagio in G minor”,  measures 9-26 | **-** |
| Samuel Barber “Adagio for Strings”  (Op. 11), measures 1-10 | **-** |
| Max Bruch "Kol Nidrei“  (Op. 47), measures 10-23 | entrance of cello and orchestra (measure 21) |
| Fr$é$d$é$ric Chopin “Piano Concerto No. 1”  (Op. 11), 2^nd^ movement, measures 13 – 30 | crescendo and chromatic progression (measure 29) |
| Felix Mendelssohn Bartholdy “Piano Trio No. 1”(Op. 49), 2^nd^ movement, measures 1-20 | entrance of violin and viola  (measure 9) |
| Wolfgang Amadeus Mozart “Piano Concerto No. 23”(K. 488), 2^nd^ movement, measures 1-19 | changes in volume and pitch  (measure 16) |

*Harsh sounds and surrounding music pieces*

| Harsh sound | Music piece |
| --- | --- |
| fork scraping on porcelain | Max Bruch “Scottish Fantasy”  (Op. 46), 1^st^ movement, measures 8-25 |
| cutting styrofoam I | Max Bruch “Scottish Fantasy”  (Op. 46), 3^rd^movement, measures 1-23 |
| fork scraping on a clay pot | Frederic Chopin „Piano Concerto No. 2 in F minor“  (Op. 21), 2^nd^movement, measures 6-18 |
| fingernails scraping down a blackboard | Antonin Dvorak  „Dumky-Trio“  (Op. 90), 2^nd^ movement, measures 1-29 |
| fork scraping on a clay pot II | Felix Mendelssohn Bartholdy „Violin Concerto in E minor“ (Op. 64), 2^nd^ movement, measures 1-21 |
| cutting styrofoam II | Wolfgang Amadeus Mozart „Piano Concerto No. 20”  (K. 466), 2^nd^ movement, measures 17-44 |

All sounds and music were edited and cut with Audacity (Audacity, Dominic Mazzoni) and Cool Edit 2000 (Syntrillium Software Corporation, Phoenix, AZ; USA). To avoid unequal volume peaks an amplitude normalization was realized. Fade-in and fade-out effects were added to the musical excerpts in order to allow a smooth beginning and ending and, thus, to avoid strong orienting responses. All sound stimuli were stored and played in WAV format. The experimental procedure was run with Presentation software (Neurobehavioral Systems, Inc.). The twelve stimuli were presented in such a way that no more than two stimuli from the same category were played consecutively. A separate experimental procedure was conducted to ensure that pressing the grip handle alone did not elicit relevant skin conductance responses and to receive maximum grip strength values for scaling the results.

*Chill experience*

Participants were instructed to listen carefully to the music and sounds and to report intensity, onset and duration of each chill experience by pressing a foam covered handle device with their right hand. The handle device was connected to a computer via a Respiration Belt MR Transducer module and a BrainAmpExG MR device (all Brain Products, Gilching, Germany) which converted pressure changes caused by pressing the handle to a digital signal. Recording was realized with BrainVision Recorder software (Brain Products, Gilching, Germany) in arbitrary units. Sampling rate was set at 5000Hz.

*Skin conductance*

Skin conductance was recorded with two Ag/AgCl electrodes (Brain Products, Gilching, Germany) with a diameter of 45-mm and filled with a 0.05 M sodium chloride electrolyte medium placed adjacently on the hypothenar eminence of the left hand. Connected to a GSR-MR module and a BrainAmpExG MR device the signal was amplified and recorded with BrainVision Recorder software (all Brain Products, Gilching, Germany). Sampling rate was set at 5000Hz.

*MRI*

During each of the two runs, 864 volumes with 34 slices (1mm gap) were acquired using echo-planar imaging (TR , 2000ms; TE 30 ms; flip angle 70°; FOV 192x192mm²; matrix size 64 x 64, voxel size 3 x 3 x 3 mm³). The first three volumes in each run were discarded to allow for T1 equilibration effect. Thirty-four phase and magnitude images were acquired in the same FOV by a gradient echo sequence (flip angle 60◦; FOV 192×192; slice thickness 3 mm; TR 488 ms; TE1 4.92 ms; TE2 7.38 ms) to calculate a fieldmap aiming at correcting geometric distortions in the EPI images (Hutton et al., 2002). T1-weighted structural scans were also acquired (MP-RAGE, TR 1690, TE 2.52 ms, flip angle 9°, matrix size 256 x 256, voxel size 1x1x1 mm³). The whole measurement duration was about 7 minutes. For diffusion weighted imaging we applied a Siemens MDDW (Multi Directional Diffusion Weighting) sequence with the following parameter setup: voxel size: 1.8 × 1.8 × 2.3mm^3^, 55 slices, 1 acquisition and 64 directions. One b0-volume was measured and b=1000 s/mm^2^ was used for the diffusion-weighted images. TR was 10500ms, TE: 107ms and the total scan time was 12 minutes.

***Imaging Processing and Statistical Analyses***

*Ratings of Chill Experiences*

Data of the handle device were processed with Brain Vision Analyzer 2.0 (Brain Products, Gilching, Germany). A signal increase caused by pressing the handle was assessed via a built-in peak finder function. Each peak was then treated as a chill report with the peak value as its reported intensity. To cope with slight baseline pressure changes, which occurred over the course of the experiment, intensity values of the reported chills were adjusted with regard to the baseline pressure of the corresponding section. Since grip strength can differ among humans, the scored intensity values were then range corrected by dividing each intensity value by the individual maximum grip strength shown in a separate experimental procedure. Numbers and means of the range corrected intensities were computed for chill reports in response to music and harsh sounds. Chills reported before the onset or after the offset of the harsh sounds were not considered for calculations.

*Skin Conductance Change*

Skin conductance data were preprocessed using Brain Vision Analyzer 2.0 (Brain Products, Gilching, Germany), down-sampled to 10Hz. Further calculations were then realized with Ledalab software package (http://www.ledalab.de) which applies a deconvolution of the raw skin conductance data into tonic and phasic activity. This was done in order to overcome drawbacks of the classic through-to-peak method such as an underestimation of phasic peaks (Benedek & Kaernbach, 2010). Analysis of skin conductance response (SCR) was focused on chill passages which were defined as 10s windows starting either with the onset of the harsh sounds (unpleasant chill hotspot) or with the beginning of specific passages in the music stimuli (pleasant chill hotspot). SCR was then scored as the average phasic activity (Windel et al., 2015) in a 9s window starting one second after the onset of the chill passage. A logarithmic transformation of the SCR values was carried out to normalize the distribution. In order to examine the relationship between the subjective chill experience and the associated physiological arousal the SCR values were then correlated with the reported chill intensity for their respective chill passage. In case of multiple chills reported closely one after another the highest reported intensity was considered for calculations. When no chill was reported for a chill passage, the intensity value was set to 0. Since the excerpts by Albinoni and Barber did not contain particular acoustical changes (Guhn et al., 2007), they were not considered. Hence, 10 chill passages were included in the correlation analysis. Analogously, SCRs were also correlated with BOLD magnitude. Skin conductance level (see Figure 3) was calculated from the raw skin conductance data in half-second bins.

*MRI data*

Lesion volumes were calculated by manually drawing the border of the lesion in the high-spatial-resolution T1-weighted image for each slice and by estimating the resulting volume (cc) with MRIcron (<http://www.sph.sc.edu/comd/rorden/mricron>).

Diffusion weighted data were corrected for eddy current and head movement artifacts and further processed utilizing DTIFIT, BEDPOSTX and PROBTRACKX of the FSL software package. DTIFIT was used to calculate the diffusion tensor and fractional anisotropy (FA). BEDPOSTX was executed to build up distributions on diffusion parameters and modeling crossing fibers at each voxel of the brain. PROBTRACKX was used to calculate a structural connectivity distribution between selected regions-of-interest (Behrens et al., 2007). The resulting probabilistic streamlines were then normalized by region-of-interest sizes (volume of all contained voxels) and thresholded with 10% of the highest connectivity value of a certain probabilistic streamline. The normalized and thresholded probabilistic streamline was evaluated with regard to its weighted mean fractional anisotropy.

Seed masks for tractography were created from the activation maxima of the fMRI statistical maps within the PFC using a spherical kernel with a diameter of 15mm to ensure the inclusion of white matter. The resulting file was filtered by a binary white matter mask in order to remove every non-white matter voxel data. Additional seed masks for the evaluation of the arcuate fasciculus, cingulate gyrus, the posterior limb of the internal capsule, the genu of the Corpus Callosum and the Corona radiata were taken from the JHU ICBM-DTI-81 White-Matter Labels (Mori et al., MRI Atlas of Human White Matter. Elsevier, Amsterdam, The Netherlands (2005)). The final seed masks were then spatially transformed from MNI space into each specific subject space, in order to avoid tractography errors by normalization (Domin et al., 2014).

FMRI data were analyzed using the SPM 8 software (Wellcome Department of Cognitive Neurosciences, London, http://www.fil.ion.ucl.ac.uk./spm) running under Matlab (Math Works; Natick, MA). Spatial pre-processing included realignment to the first scan, unwarping, coregistration to the T1 anatomical volume images. Unwarping of geometrically distorted EPIs was performed using the Field Map Toolbox. T1 images were segmented into gray and white matter as well as the cerebro-spinal fluid, bias corrected and spatially normalized using the New Segment function of SPM 8. A template normalized image was created using DARTEL (Ashburner, 2007) and functional and T1-weighted images were normalized to this template into the MNI-space. Normalized functional images were smoothed using SPM 8’s smoothing function with a Gaussian kernel of 6 × 6 × 6 mm^3^ full-width at half-maximum to increase the signal to noise ratio and reduce inter-subject differences. FMRI data were analyzed using a general linear model (GLM) with chill condition versus baseline condition (bird song). At the first level of analysis, a fixed-effect analysis was performed to obtain fMRI-activation maps for each subject due to pleasant and unpleasant stimuli. Therefore the harsh sound events were modeled with durations of 10s after stimulus onset and compared to baseline events. The pleasant chill events were modeled separately and onsets were chosen individually for MC and HC based on the device pressure and events were modeled 10s around this time point. Again a comparison with baseline was conducted. We used a statistical threshold of p< .05 corrected for multiple comparisons in the whole brain volume (family wise error; FWE).

For HC we further conducted two psychophysiological interaction (PPI) analyses with SPM to investigate which voxels in the brain increase their interaction with the left and the right AIC during a chill event. Therefore the convoluted time courses (task × seed) were analyzed in a GLM while the task time course and the seed time course itself were included as regressors. The task time course was modeled with both types of chill events together. The seed regions were 6mm spheres at (x,y,z)= -42,15,-9 for left AIC and (x,y,z)= 45,12,-6 for right AIC and results were thresholded for p_FWE_< .05 for prefrontal ROIs (BAs 8/9, 44/45; 46/47) and Heschl’s gyrus.

***References Supplement***

Ashburner, J. (2007). A fast diffeomorphic image registration algorithm. NeuroImage, 38(1), 95–113.

Benedek, M. & Kaernbach, C. (2010). A continuous measure of phasic electrodermal activity. Journal of Neuroscience Methods, 190(1), 80–91.

Domin, M., Langner, S., Hosten, N., Lotze, M. (2014). Comparison of Parameter Threshold Combinations for Diffusion Tensor Tractography in Chronic Stroke Patients and Healthy Subjects. PLoS ONE, 9(5), e98211.

Ely, D. J. (1975). Aversiveness without pain: Potentiation of imaginai and auditory effects of blackboard screeches. Bulletin of the Psychonomic Society, 6(3), 295–296.

Guhn, M., Hamm, A., & Zentner, M. (2007). Physiological and Musico-Acoustic Correlates of the Chill Response. Music Perception, 24(5), 473–484.

Reuter, C., & Oehler, M. (2011). Psychoacoustics of chalkboard squeaking. The Journal of the Acoustical Society of America, 130(4), 2545.

Windel, A. S., Mihai, P. G., & Lotze, M. (2015). Neural representation of swallowing is retained with age. A functional neuroimaging study validated by classical and Bayesian inference. Behavioural Brain Research, 286, 308–317.
